# Outcomes in elderly Chinese patients with atrial fibrillation and coronary artery disease. A report from the Optimal Thromboprophylaxis in Elderly Chinese Patients with Atrial Fibrillation (ChiOTEAF) registry

**DOI:** 10.1002/joa3.12744

**Published:** 2022-05-31

**Authors:** Agnieszka Kotalczyk, Yutao Guo, Ameenathul M. Fawzy, Yutang Wang, Gregory Y. H. Lip

**Affiliations:** ^1^ Liverpool Centre for Cardiovascular Science University of Liverpool and Liverpool Heart & Chest Hospital Liverpool UK; ^2^ Department of Cardiology, Congenital Heart Diseases and Electrotherapy Medical University of Silesia, Silesian Centre for Heart Diseases Zabrze Poland; ^3^ Department of Pulmonary Vessel and Thrombotic Disease, Sixth Medical Centre Chinese PLA General Hospital Beijing China; ^4^ Department of Cardiology, Second Medical Centre Chinese PLA General Hospital Beijing China; ^5^ Department of Clinical Medicine Aalborg University Aalborg Denmark

**Keywords:** Asia, atrial fibrillation, coronary artery disease, elderly, oral anticoagulation

## Abstract

**Background:**

Atrial fibrillation (AF) and coronary artery disease (CAD) are closely related; CAD may precede or complicate the clinical course of AF.

**Objective:**

To evaluate the impact of CAD on clinical outcomes among elderly Chinese AF patients.

**Methods:**

The ChiOTEAF registry is a prospective registry conducted in 44 sites from 20 provinces in China between October 2014 and December 2018. Primary outcome was the composite of all‐cause mortality/any thromboembolism (TE)/major bleeding/acute coronary syndrome (ACS).

**Results:**

The eligible cohort for this analysis included 6403 individuals (mean age 74.8 ± 10.7; 39.2% female); of these, 3058 (47.8%) had a history of CAD. On multivariate analysis, CAD was independently associated with a higher odds ratio for ACS (OR: 1.98; 95% CI: 1.12–3.52) without a significant impact on other adverse outcomes. Independent variables associated with the composite outcome among CAD patients were: (i) the use of OAC (OR: 0.55; 95% CI: 0.42–0.72), age (OR: 1.09; 95% CI: 1.08–1.11), heart failure (OR: 1.95; 95% CI: 1.51–2.50), prior ischemic stroke (OR: 1.29; 95% CI: 1.02–1.64), chronic kidney disease (OR: 1.71; 95% CI: 1.32–2.22), and chronic obstructive pulmonary disease (OR: 1.42; 95% CI: 1.06–1.89).

**Conclusions:**

AF patients with CAD were at an increased risk of developing ACS but there was no significant difference in the composite outcome, all cause death, cardiovascular death, thromboembolic events or major bleeding compared to the non‐CAD group. OAC use was inversely associated with adverse events, yet their uptake was poor in the AF‐CAD population.

## Graphical abstract

Anticoagulant use was poor in atrial fibrillation patients with coronary artery disease (AF‐CAD). Anticoagulant use in elderly patients was protective against the composite outcome. AF‐CAD was independently associated with acute coronary syndrome.

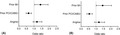

## 1 INTRODUCTION

There is a bidirectional relationship between coronary artery disease (CAD) and atrial fibrillation (AF), with multiple shared risk factors. CAD may precede or complicate the clinical course of AF.^1^ Indeed, AF‐related systemic inflammation may promote a pro‐thrombotic state and ultimately lead to myocardial infarction (MI).^2^ Previous studies have shown that AF was associated with a 70% increase in the risk of MI, but gender and race differences have been emphasized.^3^, ^4^

On the other hand, about a quarter of the AF population have a history of MI; indeed, MI is considered a risk factor for AF and AF‐related complications.^1^ Furthermore, CAD is associated with a higher incidence of thromboembolic (TE) events, even in patients without AF.^5^, ^6^ The management of AF patients with previous MI or revascularization has attracted much attention because of the complexity of antithrombotic treatment in this setting.^7^, ^8^, ^9^, ^10^, ^11^

As data among Asian patients are still sparse, we aimed to evaluate the coexistence and impact of CAD on clinical outcomes among elderly Chinese AF patients enrolled in the nationwide, prospective registry.

## 2 METHODS

The Optimal Thromboprophylaxis in Elderly Chinese Patients with Atrial Fibrillation (ChiOTEAF) registry is a prospective cohort study conducted between October 2014 and December 2018 in 44 sites from 20 Chinese provinces. A detailed description of the study design has been previously published.^12^ Briefly, consecutive AF patients presenting to cardiologists, neurologists, or surgeons were enrolled. Follow‐up visits were performed at 6 and 12 months and then annually for the following 2 years. Data were gathered by local investigators at enrollment and follow‐up visits and reported into an electronic form.

The registry was approved by the Central Medical Ethics Committee of Chinese PLA General Hospital, Beijing, China (approval no S2014‐065‐01) and local institutional review boards.

### 2.1 Definitions

Variables included in the registry and their definitions were designed to match the EORP‐AF Long‐term General Registry.^13^ CAD was categorized as either a history of acute coronary syndromes (ACS) or chronic coronary syndromes managed pharmacologically or “prior revascularization” (defined as percutaneous coronary intervention or coronary artery bypass graft).^14^ “Myocardial infarction” was defined as MI with ST‐segment elevation or MI without ST‐segment elevation. “Angina” was defined as chronic coronary syndromes with anginal symptoms. The CHA_2_DS_2_‐VASc score^15^ and the HAS‐BLED bleeding score^16^ were used to assess the thromboembolic (TE) and bleeding risks. Bleeding events were categorized according to the ISTH definition.^17^

### 2.2 Objectives

The principal objectives of the present analysis were as follows: (i) to describe the baseline characteristics and the incidence of adverse events at 1‐year follow‐up among AF patients with CAD; (ii) to evaluate the impact of CAD on clinical outcomes, including the composite outcome of all‐cause death/any TE (ischemic stroke, transient ischemic attack, or peripheral embolism)/major bleeding/ACS (MI with ST‐segment elevation, MI without ST‐segment elevation, unstable angina); and (iii) to identify potential predictors of the composite outcome in patients with CAD.

The secondary objectives were: (i) to evaluate the impact of CAD on all‐cause death, cardiovascular death, TE events, major bleeding, and ACS; and (ii) to assess the effect of prior MI, angina, and prior revascularization on the composite outcome and all‐cause death among the CAD group.

### 2.3 Statistical analysis

Continuous variables were reported as the mean ± standard deviation (SD); between‐group comparisons were made using the Student's *t*‐test or the Mann–Whitney U test (based on distribution). Categorical variables as counts and percentages; between‐group comparisons were made by χ^2^ test. A logistic univariate regression analysis was used to evaluate the potential association between CAD and clinical outcomes, as well as to evaluate the predictors of the composite outcome and all‐cause death in the CAD group. Results were expressed as odds ratios (ORs), 95% confidence intervals (CIs), and P‐values. All the significant variables of relevant clinical interests were subsequently included in a multivariate regression model.

In all analyses, a value of *p* < .05 was considered statistically significant. Statistical analysis was performed using SPSS® version 24 (IBM Corp).

## 3 RESULTS

**FIGURE 1:**
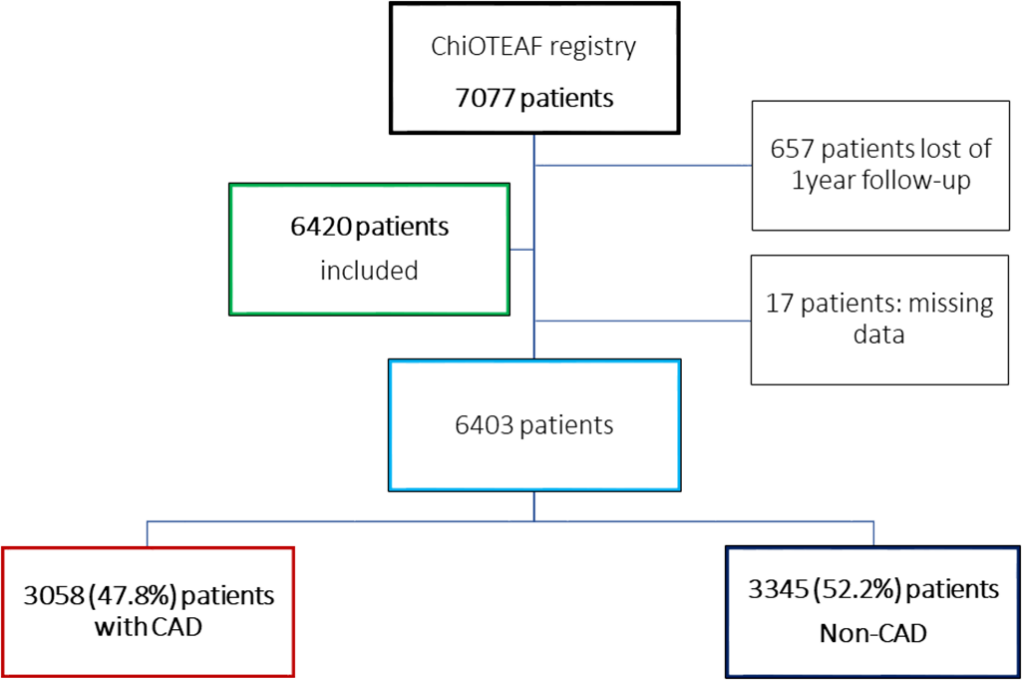
Flowchart of patient inclusion. CAD, coronary artery disease; ChiOTEAF, Optimal thromboprophylaxis in Elderly Chinese Patients with Atrial Fibrillation.

The ChiOTEAF registry enrolled 7077 patients, of whom 657 (9.3%) were lost to follow‐up at 1‐year (Figure 1). The eligible cohort for this analysis included 6403 individuals (mean age 74.8 ± 10.7; 39.2% female); of these, 3058 (47.8%) had a history of CAD (AF‐CAD patients; CAD group), and 3345 (52.2%) had no previous diagnosis of CAD (non‐CAD group). Baseline characteristics are reported in Table 1.

**TABLE 1:** Baseline characteristics of study cohort

	Overall *N* = 6403 *n* (%)	CAD *N* = 3058 *n* (%)	Non‐CAD *N* = 3345 *n* (%)	*p*‐value
Age*; years	74.8 ± 10.7	77.5 ± 10.0	72.3 ± 10.6	<.001
Female gender	2509 (39.2)	1156 (37.8)	1353 (40.4)	.030
BMI*, kg/m^2^	24.1 ± 3.6	24.2 ± 3.6	24.1 ± 3.7	.419
First diagnosed AF (*n* = 5494)	948 (17.3)	461 (18.3)	487 (16.4)	.063
Medical history				
Diabetes	1682 (26.3)	1003 (32.8)	679 (20.3)	<.001
Lipid disorder	2800 (43.7)	1801 (58.9)	999 (29.9)	<.001
Hypertension	4073 (63.6)	2177 (71.2)	1896 (56.7)	<.001
Heart failure	2290 (35.8)	1507 (49.3)	783 (23.4)	<.001
LVEF* (%) (*n* = 4592)	58.6 ± 9.6	57.3 ± 10.1	59.9 ± 8.9	<.001
Prior ischemic stroke	1587 (24.8)	955 (31.2)	632 (18.9)	<.001
Prior major bleeding (*n* = 6396)	265 (4.1)	150 (4.9)	115 (3.4)	.003
Chronic kidney disease	790 (12.3)	511 (16.7)	279 (8.3)	<.001
COPD	598 (9.3)	377 (12.3)	221 (6.6)	<.001
Liver disease	256 (4.0)	131 (4.3)	125 (3.7)	.265
Peripheral artery disease	527 (10.9)	350 (15.5)	177 (6.8)	<.001
Sleep apnea	213 (3.3)	122 (4.0)	91 (2.7)	.005
Dementia	223 (3.5)	147 (4.8)	76 (2.3)	<.001
Smoking Former smoker Current smoker	1232 (19.4) 491 (7.7)	667 (22.1) 210 (7.7)	210 (7.0) 281 (8.5)	<.001 .026
CHA_2_DS_2_VASC* (*n* = 5925)	3.6 ± 1.7	4.2 ± 1.7	3.1 ± 1.6	<.001
HAS‐BLED* (*n* = 6031)	2.15 ± 1.1	2.5 ± 1.1	1.8 ± 1.0	<.001
Medications				
OAC (*n* = 6399)	2797 (43.7)	1112 (36.4)	1685 (50.4)	<.001
Warfarin	1343 (21.0)	540 (17.7)	803 (24.0)	<.001
NOAC	1454 (22.7)	572 (18.7)	882 (26.4)	<.001
Aspirin (*n* = 6389)	1825 (28.6)	1268 (41.5)	557 (16.7)	<.001
Clopidogrel (*n* = 6389)	1269 (19.9)	955 (31.3)	314 (9.4)	<.001
Ticagrelor	25 (0.4)	15 (0.5)	10 (0.3)	.220
ACEI	841 (13.2)	478 (15.7)	363 (10.9)	<.001
ARB	1644 (25.8)	847 (27.8)	797 (23.9)	<.001
β‐blocker	3385 (53.1)	1864 (61.3)	1521 (45.6)	<.001
Statins	3603 (56.4)	2226 (73.1)	1377 (41.3)	<.001
Digoxin (*n* = 6383)	751 (11.8)	386 (12.7)	365 (10.9)	.032
Amiodarone (*n* = 6386)	914 (14.3)	391 (12.8)	523 (15.7)	<.001
Propafenone (*n* = 6386)	291 (4.6)	70 (2.3)	221 (6.6)	<.001
Diuretics (*n* = 6383)	1811 (28.4)	1078 (35.4)	733 (22.0)	<.001
Calcium channel blockers (*n* = 6382)	1715 (26.9)	922 (30.3)	793 (23.8)	<.001
Nitrates (*n* = 6388)	1569 (24.6)	1243 (40.7)	326 (9.8)	<.001

a Mean ± standard deviation.

*Note*: ACE‐I, angiotensin‐converting enzyme inhibitor; AF, atrial fibrillation; ARB ‐ angiotensin II receptor blocker; BMI, body mass index; CHA_2_DS_2_VASc: Congestive heart failure or left ventricular dysfunction, Hypertension, Age ≥75 (doubled), Diabetes, Stroke (doubled), Vascular disease, Age 65–74, female Sex; COPD: chronic obstructive pulmonary disease; COPD, chronic obstructive pulmonary disease; HAS‐BLED: Hypertension, Abnormal renal/ liver function, Stroke, Bleeding history or predisposition, Labile international normalized ratio, Elderly, Drugs/alcohol use; LVEF, left ventricular ejection fraction; NOAC, non‐vitamin K antagonist oral anticoagulant; OAC, oral anticoagulation; VKA, vitamin K antagonist.

AF‐CAD patients were older (mean age 77.5 ± 10.0 vs. 72.3 ± 10.6; *p* < .001), with a higher incidence of co‐morbidities, consequently had higher risks of stroke (mean CHA_2_DS_2_VASc score 4.2 ± 1.7 vs. 3.1 ± 1.6; *p* < .001) and bleeding (mean HAS‐BLED score 2.5 ± 1.1 vs. 1.8 ± 1.0; *p* < .001) compared with the non‐CAD group.

**FIGURE 2:**
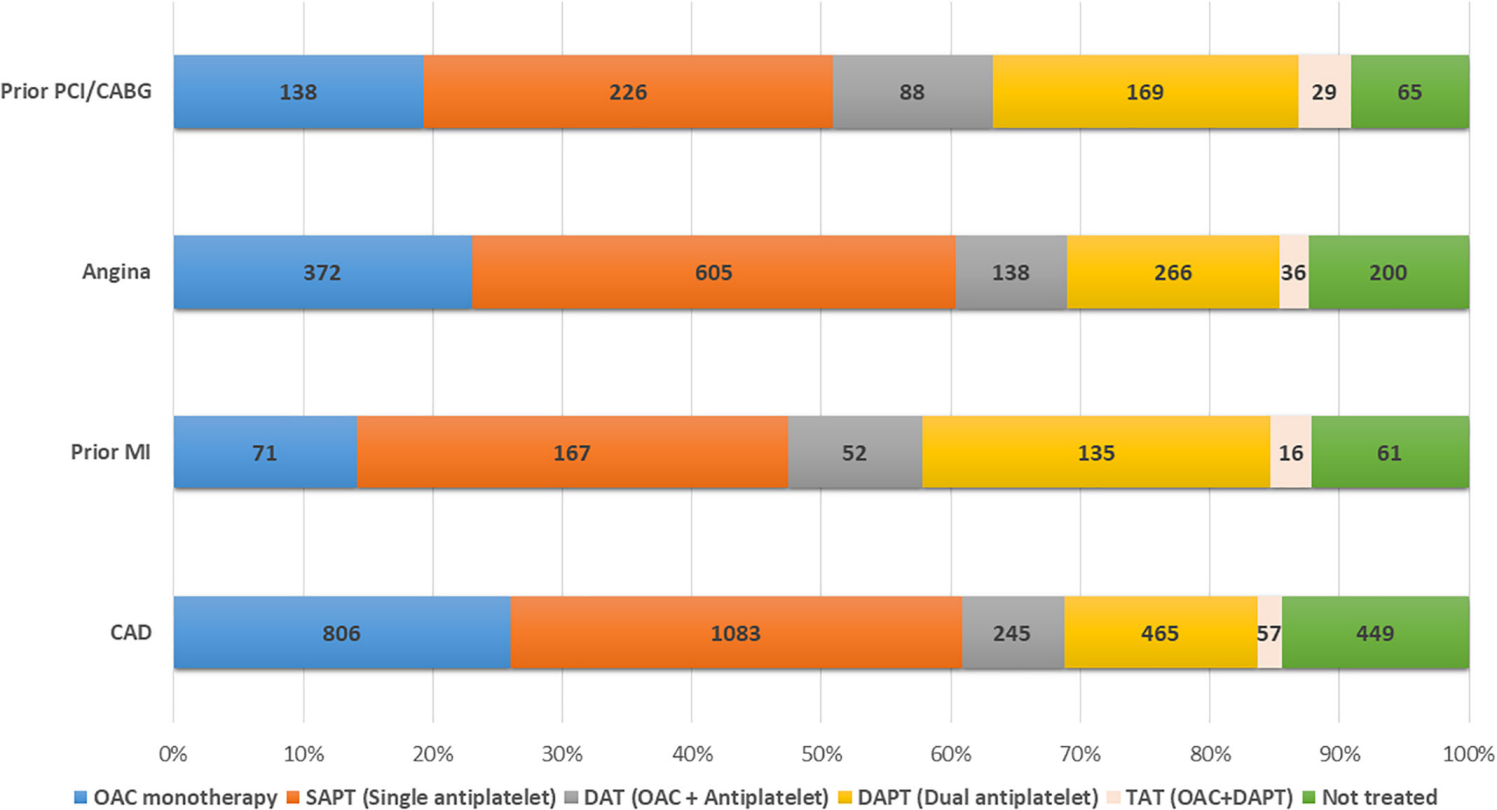
Antithrombotic strategies among patients in the coronary artery disease group. CAD, coronary artery disease; CABG, coronary artery bypass graft; DAPT, dual antiplatelet agent; DAT, dual antithrombotic therapy; MI, myocardial infarction; OAC, oral anticoagulation; PCI, percutaneous intervention; SAPT, single antiplatelet agent; TAT, triple antithrombotic therapy.

In the CAD group, 1620 (53.0%) had angina, 502 (16.4%) had previous MI, and 717 (23.6%) had revascularization. CAD patients were less likely treated with an OAC compared with non‐CAD group (36.4% vs. 50.4%; *p* < .001). Among a subgroup of patients with previous MI, only 139 (27.7%) were treated with OAC and the vast majority was treated with antiplatelet therapy (Figure 2).

### 3.1 Mortality and morbidity

AF‐CAD patients had higher rates of the composite outcome (12.5% vs. 6.5%; *p* < .001), all‐cause death (9.0% vs. 4.8%; *p* < .001), cardiovascular death (2.7% vs. 1.2%; *p* < .001), TE events (2.0% vs., 1.2%; *p* = .005), major bleeding (2.2% vs. 0.9%; *p* < .001), and ACS (1.6% vs. 0.5%; *p* < .001) compared with the non‐CAD group.

On multivariate analysis, CAD was independently associated with a higher odds ratio for ACS (OR: 1.98; 95% CI: 1.12–3.52) without a statistically significant impact on other adverse outcomes – Table 2.

**TABLE 2:** Risk of clinical outcomes in patients with coronary artery disease (CAD) versus patients without coronary artery disease (non‐CAD) during 1‐year follow‐up

Outcomes	Overall *N* = 6403 *n* (%)	CAD *N* = 3058 *n* (%)	Non‐CAD *N* = 3345 *n* (%)	*p*	Univariate odds ratio (95% CI)	Multivariate Odds ratio* (95% CI)
Composite outcome (*n* = 6400)^b^	598 (9.3)	382 (12.5)	216 (6.5)	<.001	2.07 (1.74–2.46)	0.99 (0.82–1.22)
All‐cause death	435 (6.8)	275 (9.0)	160 (4.8)	<.001	1.97 (1.61–2.41)	0.83 (0.66–1.04)
CV death	124 (1.9)	84 (2.7)	40 (1.2)	<.001	2.33 (1.59–3.41)	1.14 (0.76–1.71)
TE events (*n* = 6368)	101 (1.6)	62 (2.0)	39 (1.2)	.005	1.76 (1.18–2.64)	1.06 (0.69–1.63)
Major bleeding (*n* = 6368)	98 (1.5)	67 (2.2)	31 (0.9)	<.001	2.41 (1.57–3.69)	1.43 (0.91–2.26)
ACS (*n* = 6368)	66 (1.0)	48 (1.6)	18 (0.5)	<.001	2.96 (1.72–5.10)	**1.98 (1.12–3.52)**

a adjusted for age, sex, oral anticoagulation, prior ischemic stroke, chronic kidney disease, heart failure.

^b^ Composite outcome of all‐cause death/any thromboembolism/acute coronary syndrome/major bleeding.

Abbreviation: ACS, acute coronary syndrome, CAD, coronary artery disease; CI, confidence interval; CV, cardiovascular; TE, thromboembolic.

### 3.2 Multivariate analysis

On multivariate analysis (Table 3), independent variables associated with the composite outcome among CAD patients were as follows: (i) the use of OAC (OR: 0.55; 95% CI: 0.42–0.72) was inversely associated; and (ii) age (OR: 1.09; 95% CI: 1.08–1.11), heart failure (OR: 1.95; 95% CI: 1.51–2.50), prior ischemic stroke (OR: 1.29; 95% CI: 1.02–1.64), chronic kidney disease (OR: 1.71; 95% CI: 1.32–2.22), and chronic obstructive pulmonary disease (OR: 1.42; 95% CI: 1.06–1.89) were associated with a higher incidence of the composite outcome.

**TABLE 3:** Predictors of the composite outcome among patients with atrial fibrillation and coronary artery disease

	Univariate			Multivariate		
	Odds ratio	95% CI	*p*‐value	Odds ratio	95% CI	*p*‐value
Age	1.12	1.10–1.14	<.001	1.09	1.08–1.11	<.001
Female gender	0.68	0.54–0.85	.001	–	–	–
Diabetes mellitus	1.37	1.09–1.70	.006	–	–	–
Hypertension	0.97	0.77–1.23	.821			
Heart failure	2.81	2.22–3.54	<.001	1.95	1.51–2.50	<.001
Prior ischemic stroke	1.82	1.46–2.26	<.001	1.29	1.02–1.64	.037
Chronic kidney disease	2.84	2.24–3.61	<.001	1.71	1.32–2.22	<.001
COPD	2.91	2.24–3.78	<.001	1.42	1.06–1.89	.018
Sleep apnea	1.14	0.68–1.92	.625			
Liver disease	1.88	1.21–2.91	.005	–	–	–
Peripheral artery disease	1.19	0.86–1.63	.299			
Prior major bleeding	2.42	0.28–20.79	.422			
OAC	0.44	0.34–0.56	<.001	0.55	0.42–0.72	<.001
Antiplatelet	0.82	0.66–1.02	.079			

Abbreviations: CI, confidence interval; COPD, chronic obstructive pulmonary disease; OAC, oral anticoagulation.

Independent factors associated with a risk of all‐cause death (Data Supplement Table I) were as follows: (i) the use of OAC (OR: 0.29; 95% CI: 0.20–0.42) and antiplatelet agents (OR: 0.39; 95% CI: 0.29–0.53) were inversely associated; and (ii) age (OR: 1.12; 95% CI: 1.10–1.14), heart failure (OR: 2.79; 95% CI: 2.03–3.84), and chronic kidney disease (OR: 2.05; 95% CI: 1.53–2.77) were associated with a higher mortality among CAD patients.

### 3.3 Exploratory analysis

**FIGURE 3:**
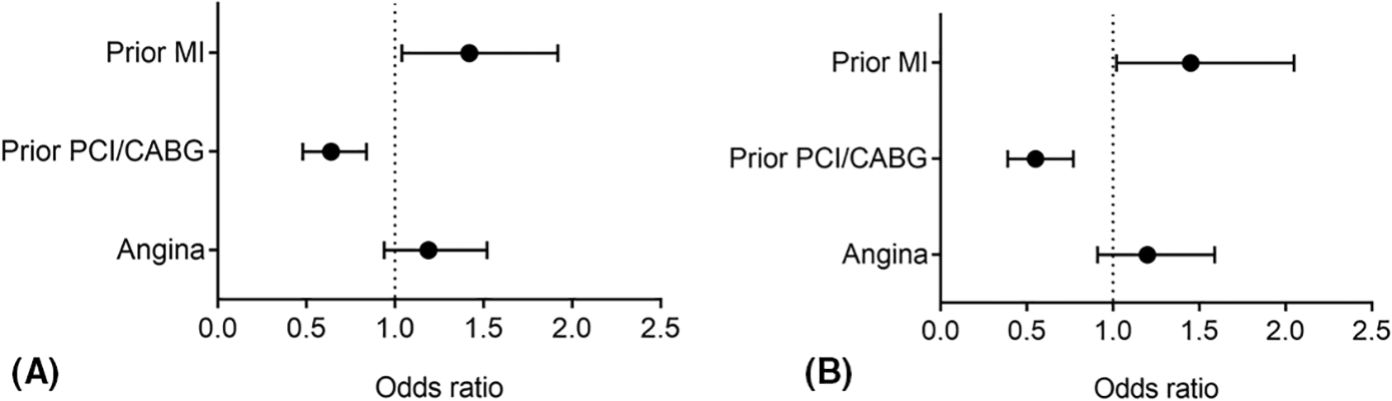
Effect of prior myocardial infarction, angina, and prior revascularization on (A) the composite outcome and (B) all‐cause death among patients with atrial fibrillation and coronary artery disease. CABG, coronary artery bypass graft; PCI, percutaneous intervention.

Prior MI, but not angina, had an additive adverse impact on the composite outcome (OR: 1.42; 95% CI: 1.04–1.92) and all‐cause death (1.45; 95% CI: 1.02–2.05) among AF patients with CAD. As expected, prior revascularization had lower odds of the composite outcome (OR: 0.64; 95% CI: 0.48–0.84) and all‐cause death (OR: 0.55; 95% CI: 0.39–0.77) among CAD patients (Figure 3; Data Supplement Table SII).

## 4 DISCUSSION

To date, few studies have looked at clinical outcomes in AF patients with CAD, particularly in the elderly Asian population. Our study demonstrates a 47.8% prevalence of CAD among AF patients, a considerably higher number compared with large‐scale international registries (approximate prevalence of 36%).^13^, ^18^ This may be explained by the older age of the participants in this study, and possibly higher prevalence of other risk factors for CAD within the study population.

In our study, AF‐CAD patients were older, with more co‐morbidities such as hypertension, heart failure, CKD and previous stroke and therefore conferred higher ischemic and bleeding risks as depicted by the mean CHA_2_DS_2_VASc and HASBLED scores compared to patients without CAD. Despite this, only 36.4% of this cohort were prescribed OAC either as monotherapy or alongside an antiplatelet agent, with rates only marginally better in the non‐CAD group (50.4%). The vast majority of CAD patients were treated with single (SAPT) or dual antiplatelet therapy (DAPT). Given the lack of precise data on the timing of prior MI or revascularization which could have ranged from years before to until the day before recruitment, it is difficult to elucidate whether the antiplatelet drug regimen was appropriate for that given point in time.

Existing guidelines recommend commencing OAC immediately in settings of ACS or revascularization, alongside SAPT or DAPT, with the latter only recommended for maximum period of 6 months in patients with very high risk of thrombosis.^19^, ^20^ This is in view of results from pivotal trials such as WOEST, ISAR‐TRIPLE, and PIONEER‐AF‐PCI which demonstrated that dual therapy (SAPT and OAC) was associated with reduction in major bleeding events with similar efficacy, when compared to triple therapy (DAPT and OAC).^21^, ^22^, ^23^ This is supported by the recent meta‐analysis which showed no significant increase in mortality, stroke, nonfatal MI, stent thrombosis, and a lower risk of bleeding with dual therapy, compared with triple therapy in AF‐CAD patients.^24^

The safety of guideline‐adherent OAC therapy among Chinese patients, including very elderly cohort (aged ≥85 years) has been demonstrated in our previous reports.^25^, ^26^ Herein, we show that OAC use was associated with a significant reduction in the composite outcomes even after multivariate adjustment, likely because of the net reduction of all‐cause death and TE events compared with bleeding outcomes in CAD patients. Overall, OAC prescription rates were poor across both groups but the significantly lower figures in the CAD group may be attributable to the major bleeding concerns associated with the concurrent use of SAPT or DAPT with OAC. Existing studies have demonstrated that NOACs are associated with a lower risk of major adverse cardiovascular events (MACE) and non‐fatal MI in AF patients.^27^ Furthermore, NOAC monotherapy has also been shown to be superior at reducing both ischemic and bleeding events beyond 1‐year poststent implantation when compared to NOAC+SAPT, suggesting that this may be the most effective strategy in this cohort of patients.^28^

Our study demonstrated that AF‐CAD patients had higher rates of all‐cause death, cardiovascular death, TE events, major bleeding, ACS and thus, a significantly higher composite outcome compared with non‐CAD patients. Interestingly, on multivariate analysis CAD was independently associated only with ACS but not the other adverse outcomes. Fukamachi et al also investigated adverse outcomes in anticoagulated Japanese AF‐CAD patients; CAD was associated with a significantly higher risk of cardiovascular events, with no differences in the TE events, bleeding, and mortality risks.^29^

These findings add to the debate about whether or not the presence of CAD confers an additional risk of ischemic stroke. After all, acute ischemic strokes are predominantly secondary to TE phenomena much like ACS, and both fall under the realm of “vascular disease,” sharing several risk factors and pathophysiological mechanisms.^30^, ^31^ The assumption therefore is that an increased risk in one should cause an increased risk in the other. While the presence of significant CAD at coronary angiography confers an increase in ischemic stroke risk, it is less clear if the extent of angiographically proven CAD (i.e., 1‐, 2‐, or 3‐vessel disease) is an independent risk factor for TE.^6^, ^32^

CAD has been associated with an increased risk of stroke in several studies.^8^ More recently, computed tomography has been used to demonstrate an increased risk of stroke in AF patients with incidental coronary artery calcification; an indicator for underlying CAD.^32^ In the study by Tagawa and colleagues, asymptomatic CAD was identified with a positive myocardial scintigraphy test in a quarter of patients with ischemic strokes.^33^ Conversely, the reverse has also been described with inconclusive evidence for CAD as an independent risk factor for stroke, consistent with our findings.^34^, ^35^

Much of the CAD group in our study comprised of patients with angina who may not have had an angiogram, meaning they may not fall under the strict definition of “angiographically significant CAD” that forms part of the CHA_2_DS_2_VASc score. This may also explain why prior MI but not angina was associated with a significantly elevated risk of the composite outcomes and all‐cause death in the sub‐analysis of the CAD group. Our data suggest that AF patients with a prior history of MI require more attention with closer monitoring and aggressive risk factor and symptom control, as part of the holistic or integrated care approach to AF case as advocated by current guidelines.^8^, ^36^ Adherence with such an integrated care approach has been associated with a major reduction in adverse clinical outcomes, including mortality, cardiovascular mortality, stroke and major bleeding, as well as hospitalizations.^37^, ^38^ In the study by Pastori et al. which specifically looked at AF patients with a high cardiovascular risk, noncompliance with the integrated care approach was associated with a 1.7 times increased risk of MACE for those with a 2MACE score of ≥3. Optimizing the antithrombotic strategy and cardiovascular risk factor management were the most important components for minimizing risk for this group.^38^

### 4.1 Limitations

Our study is subject to the limitations of an observational study design including effects from unaccounted confounders and a degree of bias because of potential selection, ascertainment, and treatment‐effect bias. Baseline data were used for analysis and therefore would not have accounted for any medication changes during the follow‐up period. The treatment regimens for individual patients will have varied depending on how far along they were from their index MI or revascularization procedure, making it difficult to ascertain whether they were on a suitable drug regimen. As this registry was primarily focused on AF, there was also a lack of data on factors relating to the revascularization procedures such as the number and types of stents used and history of stent thrombosis which may have influenced antiplatelet treatment decisions in some patients. Patients with no history of ACS and prior history of ACS were analyzed together because of inadequate numbers for multivariable adjustment in these smaller subgroups. Furthermore, it was difficult to ascertain if the observed thromboembolic events in the AF‐CAD was because of some individuals not being on OAC or being on subtherapeutic OAC, as data on “time in therapeutic range” were not available for patients on warfarin, rather than other factors. Lastly, the relatively short duration of follow‐up of 1 year in our analysis which may have allowed for observation of a fewer number of events. Overall, a low thromboembolic event rate was noted across both groups with a larger number of events being observed in the CAD group where the OAC prescription rate was lower. Thus, our findings are consistent with the inversely proportional relationship between thromboembolic events and OAC prescription rates. However, failure to demonstrate a statistically significant difference between the two groups may be because of the overall low event rate—meaning the study may not have been adequately powered for this specific outcome.

## 5 CONCLUSION

AF patients with CAD were at an increased risk of developing ACS but there was no significant difference in the composite outcome, all cause death, cardiovascular death, TE events, or major bleeding compared with the non‐CAD group. OAC use was associated with a lower chance of adverse events, yet their uptake was poor in the AF‐CAD population indicating that better awareness and guideline adherence is needed, with reinforcement of their safety even in older Chinese patients.

## ETHICS APPROVAL

This study was performed in line with the principles of the Declaration of Helsinki. Ethics approval was granted by the Central Medical Ethic Committee of Chinese PLA General Hospital (approval no S2014‐065‐01).

## CONSENT TO PARTICIPATE

Written informed consent was obtained from all individual participants included in the study.

## CONSENT FOR PUBLICATION

Not applicable.

Authors are responsible for correctness of the statements provided in the manuscript.

## AVAILABILITY OF DATA AND MATERIAL

The datasets used and analyzed during the current study are available from the corresponding author on reasonable request.

## CODE AVAILABILITY

Statistical analysis was performed using SPSS® version 24 (IBM Corp, Armonk, NY).

## COMPETING INTERESTS

GYHL: Consultant and speaker for BMS/Pfizer, Boehringer Ingelheim and Daiichi‐Sankyo. No fees are received personally. The other authors have no conflict of interest.

## AUTHOR CONTRIBUTIONS

This paper has not been submitted for publication to any other journal. All authors have made a significant contribution and have read and approved the final draft. Y. Guo and A. Kotalczyk and A.M. Fawzy contributed equally to design the study, interpret data, and draft the manuscript (joint first authors); Y. Wang and GYH Lip contributed in the interpretation of data, and revised the manuscript critically for important intellectual content (joint senior authors).

## FUNDING INFORMATION

The study was supported by the Beijing Natural Science Foundation, China (Z141100002114050), and Chinese Military Health Care (17BJZ08).

## Supporting information


Appendix S1
