# Supplementary material for: Outcomes in elderly Chinese patients with atrial fibrillation and coronary artery disease. A report from the Optimal Thromboprophylaxis in Elderly Chinese Patients with Atrial Fibrillation (ChiOTEAF) registry
Source: J Arrhythm. 2022 May 31;38(4):580–8. doi: 10.1002/joa3.12744 (PMC9347193; doi:10.1002/joa3.12744)
Supplement: Supplementary file 1 — Appendix S1 [file JOA3-38-580-s001.docx]

**Outcomes in elderly Chinese patients with atrial fibrillation and coronary artery disease**

**A report from the Optimal Thromboprophylaxis in Elderly Chinese Patients with Atrial Fibrillation (ChiOTEAF) registry**

Agnieszka Kotalczyk^1,2^*, Yutao Guo^1,3^*, Ameenathul M. Fawzy^1^*, Yutang Wang^4^#,

Gregory Y.H. Lip^1,2,5^#; on behalf of the ChiOTEAF Registry Investigators†

[*joint first authors; Drs Guo, Wang and Lip are joint senior authors]

^1^ Liverpool Centre for Cardiovascular Science, University of Liverpool and Liverpool Heart & Chest Hospital, Liverpool, United Kingdom

^2^ Department of Cardiology, Congenital Heart Diseases and Electrotherapy, Medical University of Silesia, Silesian Centre for Heart Diseases, Zabrze, Poland

^3^ Department of Pulmonary Vessel and Thrombotic Disease, Sixth Medical Centre, Chinese PLA General Hospital, Beijing, 100142

^4^ Department of Cardiology, Second Medical Centre, Chinese PLA General Hospital, Beijing, 100853

^5^ Department of Clinical Medicine, Aalborg University, Aalborg, Denmark

**CONTENT:**

- **Supplementary Table I** Predictors of all-cause death among patients with atrial fibrillation and coronary artery disease.
- **Supplementary Table II.** Effect of (A) prior myocardial infarction, angina, and (B) prior revascularization on the composite outcome and all-cause death among patients with atrial fibrillation and coronary artery disease.
- List of ChiOTEAF Registry Investigators.

**Supplementary Table I** Predictors of all-cause death among patients with atrial fibrillation and coronary artery disease.

|  | Univariate | | | Multivariate | | |
| --- | --- | --- | --- | --- | --- | --- |
|  | Odds ratio | 95% CI | p-value | Odds ratio | 95% CI | p-value |
| **Age** | 1.15 | 1.13-1.18 | <0.001 | 1.12 | 1.10-1.14 | <0.001 |
| Female gender | 0.72 | 0.55-0.93 | 0.014 | - | - | - |
| Diabetes mellitus | 1.32 | 1.02-1.70 | 0.034 | - | - | - |
| Hypertension | 0.90 | 0.69-1.17 | 0.421 |  |  |  |
| **Heart failure** | 4.04 | 3.01-5.43 | <0.001 | 2.79 | 2.03-3.84 | <0.001 |
| Prior ischemic stroke | 1.75 | 1.36-2.25 | <0.001 | - | - | - |
| **Chronic kidney disease** | 3.49 | 2.67-4.55 | <0.001 | 2.05 | 1.53-2.77 | <0.001 |
| COPD | 3.08 | 2.30-4.12 | <0.001 | - | - | - |
| Sleep apnea | 1.11 | 0.60-2.04 | 0.740 |  |  |  |
| Liver disease | 2.26 | 1.42-3.61 | 0.001 | - | - | - |
| Peripheral artery disease | 1.19 | 0.82-1.71 | 0.362 |  |  |  |
| Prior major bleeding | 4.93 | 0.57-42.60 | 0.147 |  |  |  |
| **OAC** | 0.36 | 0.26-0.49 | <0.001 | 0.29 | 0.20-0.42 | <0.001 |
| **Antiplatelet** | 0.71 | 0.56-0.91 | 0.008 | 0.39 | 0.29-0.53 | <0.001 |

CI – confidence interval; COPD – chronic obstructive pulmonary disease; OAC – oral anticoagulation.

**Supplementary Table II** Effect of (A) prior myocardial infarction, angina, and (B) prior revascularization on the composite outcome and all-cause death among patients with atrial fibrillation and coronary artery disease.

|  | Composite outcome# | | | All-cause death | | |
| --- | --- | --- | --- | --- | --- | --- |
| A | Odds ratio | 95% CI | p-value | Odds ratio | 95% CI | p-value |
| CAD [ref] | REF | | | REF | | |
| Prior MI | 1.42 | 1.04-1.92 | 0.025 | 1.45 | 1.02-2.05 | 0.039 |
| Angina | 1.19 | 0.94-1.52 | 0.152 | 1.20 | 0.91-1.59 | 0.198 |
| B | | | | | |  |
| CAD [ref] | REF | | | REF | | |
| Prior PCI/CABG | 0.64 | 0.48-084 | 0.002 | 0.55 | 0.39-0.77 | 0.001 |

# composite outcome of all-cause death/any thromboembolism/acute coronary syndrome/major bleeding

CAD – coronary artery disease; CI – confidence interval; MI – myocardial infarction; PCI – percutaneous intervention; CABG – coronary artery bypass graft

**ChiOTEAF Registry Investigators**

**Academic Executive Steering Committee**

Gregory Y H Lip, MD, Liverpool Centre for Cardiovascular Science, University of Liverpool and Liverpool Heart & Chest Hospital, Liverpool, United Kingdom (Co-Chair)

Xiaoying Li, MD, PhD, Department of Geriatric Cardiology, Chinese PLA General Hospital, Beijing, China (Co-Chair)

Yutang Wang, MD, PhD, Department of Geriatric Cardiology, Chinese PLA General Hospital, Beijing, China (Co-Chair)

Changsheng Ma, MD, PhD, Department of Cardiology, Center for Atrial Fibrillation, Beijing Anzhen Hospital, Capital Medical University, Beijing, China

Shu Zhang, MD, PHD, Fuwai Hospital, Chinese Academy of Medical Sciences, Beijing, China

Congxin Huang, MD, PHD, RenMin Hospital, Wuhan University, Wuhan, China

Jiefu Yang, MD, PhD, Department of Cardiology, Beijing Hospital, Chinese Academy of Medical Sciences and Peking Union Medical College, Beijing, China.

Meilin Liu, Department of Geriatrics, Peking University First Hospital, Beijing, China

**Data Management Committee**

Gregory Y H Lip, MD, Liverpool Centre for Cardiovascular Science, University of Liverpool and Liverpool Heart & Chest Hospital, Liverpool, United Kingdom

Yutao Guo, MD, PhD, Department of Pulmonary Vessel and Thrombotic Disease, Sixth Medical Centre, Chinese PLA General Hospital, Beijing, China

Guangliang Shan, PhD, Department of Epidemiology and Statistics, Institute of Basic Medical Sciences, Chinese Academy of Medical Sciences and School of Basic Medicine, Peking Union Medical College, Beijing, China

Taixiang Wu, MD, PhD, Administrator of Chinese Clinical Trial Registry, Associate Professor of Clinical Epidemiology and Evidence-Based Medicine, West China Hospital, Sichuan University

Chen Yao, PhD, Associate Director, Peking University Clinical Research Institute, Beijing, China

**Steering Committee Members**

| \| Changsheng Ma, MD, PhD \| Anzhen Hospital, Capital Medical University, Beijing \| \| --- \| --- \| \| Congchun Huang, MD, PhD \| Air Force General Hospital, Beijing \| \| Cuntai Zhang, MD, PhD \| Tongji Hospital, Tongji Medical college, Huazhong University of Science & Technology, Guangzhou \| \| Dang Aiming, MD, PhD \| Fuwai Hospital, Chinese Academy of Medical Sciences, Beijing \| \| Dawei Qian, MD, PhD \| Ji Lin Hospital, Ji Lin \| \| Fakuan Tang, MD, PhD \| PLA 309th Hospital, Beijing \| \| Fang Wu, MD, PhD \| Rui Jin Hospital, Tong University School of Medicine, Shanghai \| \| Feng Liu, MD, PhD \| First People's Hospital, Guangdong \| \| Gexin Zhu, MD, PhD \| The General Hospital, Tianjing Medical Hospital, Tianjing \| \| Guo Yutao, MD, PhD \| PLA General Hospital, Beijing \| \| Guorong Xi, MD \| Health Division of Guard Bureau, Chinese PLA General Staff Department, Beijing \| \| Heng Dou, MD, PhD \| Beijing Hospital, Beijing \| \| Hou Cuihong, MD, PhD \| Fuwai Hospital, Chinese Academy of Medical Sciences, Beijing \| \| Hua Li, MD, PhD \| The First Affiliated Hospital, Zhengzhou University, Zhejiang \| \| Hui Han, MD, PhD \| The First Affiliated Hospital, Harbin Medical University, Heilongjiang \| \| Huiliang Liu, MD, PhD \| Wujing General Hospital, Beijing \| \| Jian Kong, MD, PhD \| The First Affiliated Hospital, Ji Lin University, Ji Lin \| \| Junxia Li, MD, PhD \| Beijing PLA General Hospital, Beijing \| \| Liang Zaoguang \| The First Affiliated Hospital, Harbin Medical University, Heilongjiang \| \| Liangyi Si, MD, PhD \| Southwest Hospital, Chongqing \| \| Liu Meilin, MD, PhD \| The First Affiliated Hospital, Peking University First Hospital, Beijing \| \| Liu Yanxia, MD \| Shenyang General PLA Hospital \| \| Liu Yu, MD \| Yanggu People's Hospital, Shandong \| \| Liu Zhiming, MD, PhD \| Fuwai Hospital, Chinese Academy of Medical Sciences, Beijing \| \| Luo Ma, MD, PhD \| NAVY General Hospital, Beijing \| \| Ming Li, MD, PhD \| Beijing Friendship Hospital, Capital Medical University, Beijing \| \| Qian Xiao, MD, PhD \| First Affiliated Hospital, Chongqing Medical University, Chongqing \| \| Qingwei Chen, MD, PhD \| The Second Affiliated Hospital, Chongqing Medical University, Chongqing \| \| Qiong Chen, MD, PhD \| Xiangya Hospital, Central South University, Hunan \| \| Ren Xuejun, MD, PhD \| Anzhen Hospital, Capital Medical University, Beijing \| \| Shan Zhaoliang, MD, PhD \| PLA General Hospital, Beijing \| \| Shi Xiangming, MD, PhD \| PLA General Hospital, Beijing \| \| Shilian Hu, MD, PhD \| Anhui Provincial Hospital, Anhui \| \| Song Bai, MD, PhD \| First Affiliated Hospital of Kunming Medical University, Kunming \| \| Tianchang Li, MD, PhD \| NAVY General Hospital, Beijing \| \| Wang Lijuan, MD \| Suqian People's hospital, Jiangsu \| \| Wu Qiang, MD, PhD \| Guizhou Provincial People's Hospital \| \| Xianghu Wang, MD, PhD \| Union Hospital, Tongji Medical College, Huazhong University of Science & Technology, Guangzhou \| \| Xiaojuan Bai, MD, PhD \| Sheng Jing Hospital, China Medical University, Shengyang, Liaoning \| \| Xiaoming Wang, MD, PhD \| Xijing Hospital, Xian \| \| Xinchun Yang, MD, PhD \| Chao-Yang Hospital, Capital Medical University, Beijing \| \| Xuan He, MD, PhD \| Air Force General Hospital, Beijing \| \| Xuejun Liu, MD, PhD \| The First Affiliated Hospital, Shanxi Medical University, Shanxi \| \| Yan Li, MD, PhD \| First People's Hospital, Kunming, Yunnan \| \| Yang Jiefu, MD, PhD \| Beijing Hospital, Beijing \| \| Yong Wang, MD, PhD \| China-Japan Friendship Hospital, Beijing \| \| Yunmei Yang, MD, PhD \| The First Affiliated Hospital, Zhenjiang University, Zhejiang \| \| Zeng Yuan, MD, PhD \| PLA 306 Hospital \| \| Zhang Shu, MD, PhD \| Fuwai Hospital, Chinese Academy of Medical Sciences, Beijing \| \| Zhang Wei, MD, PhD \| Beijing PLA General Hospital, Beijing \| \| Zhanyi Lin, MD, PhD \| Guangdong General Hospital, Guangdong \| |  |
| --- | --- | --- | --- | --- | --- | --- | --- | --- | --- | --- | --- | --- | --- | --- | --- | --- | --- | --- | --- | --- | --- | --- | --- | --- | --- | --- | --- | --- | --- | --- | --- | --- | --- | --- | --- | --- | --- | --- | --- | --- | --- | --- | --- | --- | --- | --- | --- | --- | --- | --- | --- | --- | --- | --- | --- | --- | --- | --- | --- | --- | --- | --- | --- | --- | --- | --- | --- | --- | --- | --- | --- | --- | --- | --- | --- | --- | --- | --- | --- | --- | --- | --- | --- | --- | --- | --- | --- | --- | --- | --- | --- | --- | --- | --- | --- | --- | --- | --- | --- | --- | --- | --- | --- |
